# A vicious cycle of bisretinoid formation and oxidation relevant to recessive Stargardt disease

**DOI:** 10.1016/j.jbc.2021.100259

**Published:** 2021-01-07

**Authors:** Jin Zhao, Hye Jin Kim, Keiko Ueda, Kevin Zhang, Diego Montenegro, Joshua L. Dunaief, Janet R. Sparrow

**Affiliations:** 1 Department of Ophthalmology, Columbia University Medical Center, New York, New York, USA; 2 Department of Ophthalmology, University of Pennsylvania, Philadelphia Pennsylvania, USA; 3 Department of Pathology and Cell Biology, Columbia University Medical Center, New York, New York, USA

**Keywords:** retina, retinal pigment epithelium, iron, bisretinoid, lipofuscin, Fenton reaction, light, ABCA4, ATP-binding cassette, sub-family A, member 4, AMD, age-related macular degeneration, ERG, electroretinography, ONH, optic nerve head, ONL, outer nuclear layer, qAF, quantitative fundus autofluorescence, RPE, retinal pigment epithelial cells, STGD1, Stargardt Disease 1

## Abstract

The ability of iron to transfer electrons enables the contribution of this metal to a variety of cellular activities even as the redox properties of iron are also responsible for the generation of hydroxyl radicals (^•^OH), the most destructive of the reactive oxygen species. We previously showed that iron can promote the oxidation of bisretinoid by generating highly reactive hydroxyl radical (^•^OH). Now we report that preservation of iron regulation in the retina is not sufficient to prevent iron-induced bisretinoid oxidative degradation when blood iron levels are elevated in liver-specific hepcidin knockout mice. We obtained evidence for the perpetuation of Fenton reactions in the presence of the bisretinoid A2E and visible light. On the other hand, iron chelation by deferiprone was not associated with changes in postbleaching recovery of 11-*cis*-retinal or dark-adapted ERG b-wave amplitudes indicating that the activity of Rpe65, a rate-determining visual cycle protein that carries an iron-binding domain, is not affected. Notably, iron levels were elevated in the neural retina and retinal pigment epithelial (RPE) cells of *Abca4*^−/−^ mice. Consistent with higher iron content, ferritin-L immunostaining was elevated in RPE of a patient diagnosed with ABCA4-associated disease and in RPE and photoreceptor cells of *Abca4*^−/−^ mice. In neural retina of the mutant mice, reduced Tfrc mRNA was also an indicator of retinal iron overload. Thus iron chelation may defend retina when bisretinoid toxicity is implicated in disease processes.

In addition to iron being essential for oxygen transport by hemoglobin, iron is vital to the functioning of numerous other heme- and nonheme proteins (1, 2). In mitochondria iron-containing cytochrome proteins function in the synthesis of ATP by facilitating the transfer of electrons, the iron atom in the heme group being alternately oxidized and reduced (3). Other cytochromes primarily involved in the metabolism and detoxification of substrates belong to the large family of cytochrome P450 enzymes that utilize iron as a cofactor (3). Iron is also required for the activity of ribonucleoside reductase, the rate-limiting enzyme involved in the synthesis of DNA (4) and aconitase, a nonheme iron–sulfur protein is involved in the first step of the tricarboxylic acid cycle (5). In retina, the visual cycle protein RPE65 is dependent on its iron-binding domain for activity (6).

Iron is also a redox-active metal that exerts toxicity by generating highly reactive hydroxyl radicals (^•^OH) that attack proteins, lipids, and DNA. For this reason, several mechanisms operate to prevent iron overload or deficiency by regulating Fe import, storage, and export (7). For instance, the liver secretes hepcidin into the systemic circulation to reduce blood iron levels. Circulating hepcidin does this by causing the degradation of the cellular iron exporter ferroportin that is located on cell surfaces of intestinal epithelium and macrophages; loss of ferroportin attenuates iron export (8). Null mutations of hepcidin in mice are associated with progressive accumulation of iron in the retina; elevation of ferritin, the iron storage protein; increased ferroportin and subsequent photoreceptor cell degeneration (9).

We have previously shown that excessive iron in retinal pigment epithelial (RPE) cells of mice deficient in ceruloplasmin (Cp) and hephaestin (Heph) (*Cp*^*−/−*^; *Heph*^−/−^ mice) (10) is associated with toxic bisretinoid photooxidation and degradation (11). Like hephaestin, ceruloplasmin is a ferroxidase that oxidizes iron from the ferrous (Fe^2+^, 4 unpaired electrons) to ferric (Fe^3+^, 5 unpaired electrons) state so that it can be accepted by transferrin in the circulation. RPE cells are subjected to photooxidative stress because of their content of bisretinoids. Bisretinoids are a family of photoreactive vitamin A aldehyde-derived adducts that form nonenzymatically as a by-product of the visual cycle; bisretinoids accumulate in RPE cells with age. These bisretinoid fluorophores present with two-side arms, a long and a short, consisting of conjugated systems of double bonds that are oxidized by both photo-mediated processes (12, 13) and hydroxyl radical (^•^OH) production *via* the Fenton reaction (Fe^2+^ and H_2_O_2_) (11).

Here in mechanistic studies, we embarked on an examination of RPE iron overload in liver-specific (LS) hepcidin knockout mice and tested for evidence of a photo-assisted Fenton reaction using visible light (14). As noted above, in RPE cells the enzyme Rpe65 is the iron-dependent isomerohydrolase essential for the conversion of all-*trans*-retinyl ester to 11-*cis* retinaldehyde (15); as such RPE65 catalyzes the rate-limiting step of the visual cycle. Thus we also tested for an effect of deferiprone (DFP) treatment on the activity of the visual cycle protein Rpe65. Importantly we discovered that iron levels are elevated in the retina of *Abca4*^−/−^ mice and in a patient diagnosed with ABCA4-associated disease.

## Results

### Liver-specific (LS) hepcidin knockout mice

**Figure 1:**
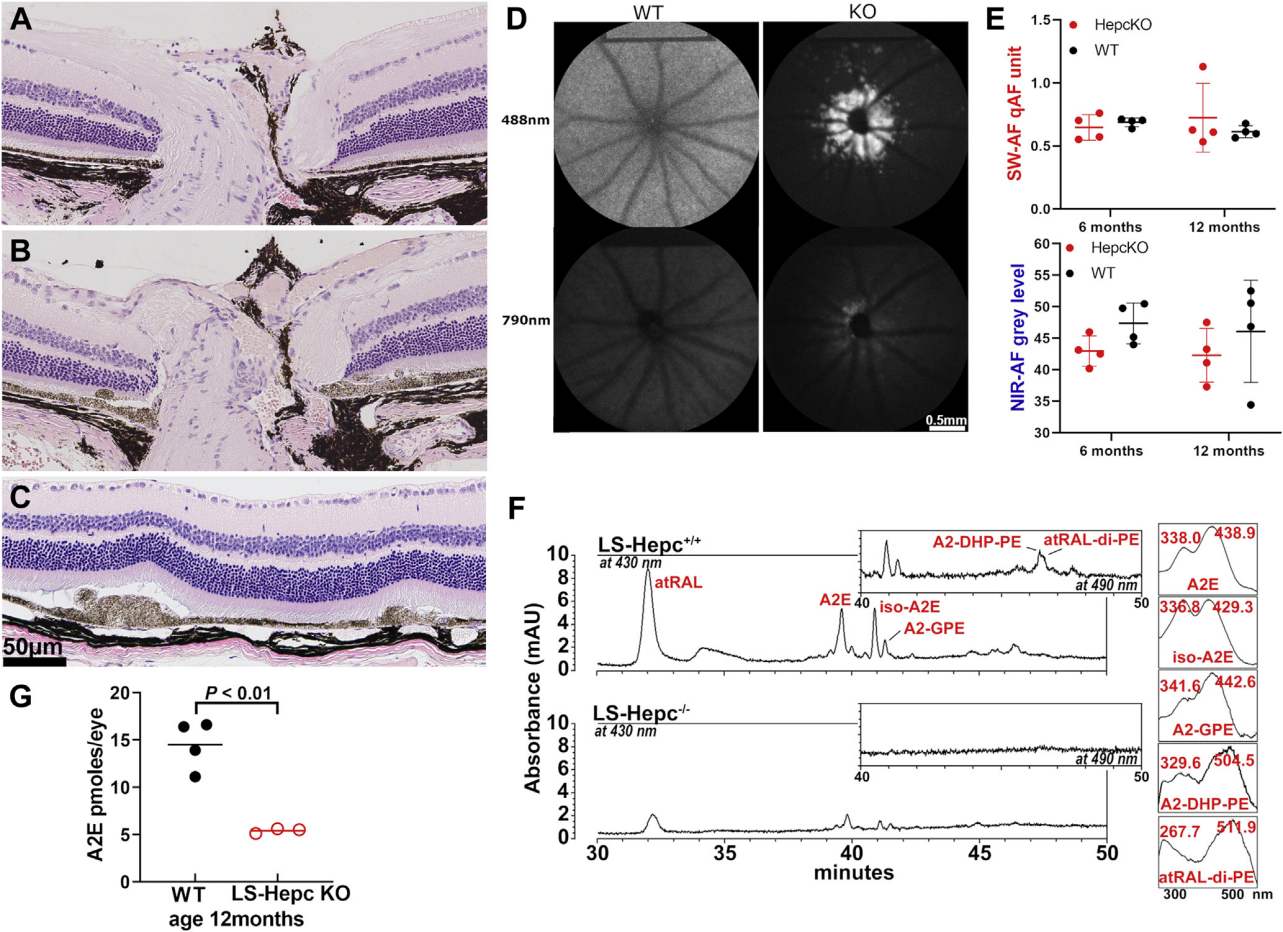
**Liver-specific (LS) hepcidin knockout (LS-Hepc**^**−/−**^**) mice.***A*–*C*, representative light micrographs of retinas acquired from wild-type mice (C57BL/6J) (*A*) and LS-Hepc^−/−^ mice (*B* and *C*) age 12 months (female). Peripapillary retina (*A* and *B*) and posterior retina (*C*). *D*, short-wavelength fundus autofluorescence (SW-AF; 488 nm excitation) and near-infrared (NIR-AF; 790 nm) fundus autofluorescence images of wild-type (WT) and LS-Hepc^−/−^ mice (KO). Age 12 months. Note the parapapillary hyperautofluorescent foci with 488 nm fluorescence. *E*, quantitation of SW-AF (quantitative fundus autofluorescence, qAF) and NIR-AF at age of 6 and 12 months. Values are means ± SD, four mice per group. *F*, HPLC quantitation of bisretinoid in liver-specific hepcidin knockout (LS-Hepc^−/−^) and wild-type (LS-Hepc^+/+^) mice (male gender). UV–visible chromatographic detection of the bisretinoids A2E, isoA2E, A2-dihydropyridine-phosphatidylethanolamine (A2-DHP-PE), all-*trans*-retinal dimer phosphatidylethanolamine (atRAL-di-PE), and A2-glycerophosphoethanolamine (A2-GPE) in LS-Hepc^+/+^ and LS-Hepc^−/−^ mice. *Insets* at right: UV–visible spectra of chromatographic peaks corresponding to the indicated bisretinoids. *G*, Picomoles of A2E per eye are plotted, age 12 months. Values are based on four (wild-type) and three (LS-Hepc^−/−^) samples (male gender), three eyes per sample. *p* value was determined by unpaired two-tailed t-test.

Hepcidin that is secreted into the bloodstream by the liver serves to reduce blood iron levels by binding to and bringing about the degradation of the iron exporter ferroportin that is expressed on the surface of cells (8). It has been shown that liver-specific Hepc knockout (LS-Hepc^−/−^) mice exhibit elevated iron in blood and increased free (labile) iron levels in the retina and RPE cells (16). In histological sections through the ONH, LS-Hepc^−/−^ mice presented with thickenings of displaced, stacked, and/or enlarged RPE cells that projected into the subretinal space at multiple locations in posterior retina; all LS-Hepc^−/−^ mice presented with these changes (Fig. 1, *B* and *C*). Within these RPE cells, melanin density was markedly reduced. At the positions of these aberrations, the RPE monolayer was discontinued, outer segments were shortened, and photoreceptor cell nuclei spanning the ONL appeared to be reduced. Nevertheless, overall ONL area (calculated as sum of thickness measurements 0.2–1.0 mm from ONH × 0.2 mm) was not different in LS-Hepc^−/−^ mice (7.59 × 10^4^ ± 0.13 μm^2^) than in wild-type mice (7.26 × 10^4^ ± 0.13 μm^2^ *p* > 0.05, t-test). An extension of the RPE monolayer at the edge of the ONH was often visible as a tuft of lightly pigmented cells (Fig. 1*A*). Atrophy of the optic nerve was not obvious, and the retinal ganglion cell layer was not changed relative to wild-type.

In addition to the histological changes, we noted a pronounced peripapillary ring of hyperautofluorescence in 4 of 8 LS-Hepc^−/−^ eyes that were imaged by SW-AF (Fig. 1*D*). This ring of AF was detectable at age 6 months and was more pronounced in images acquired at age 12 months. This peripapillary hyperautofluorescence could be indicative of increases in known fluorophores, modifications of known fluorophores, or autofluorescence from unknown fluorophores (12, 17, 18, 19, 20). Diminished autofluorescence screening by depigmented RPE at the edge of the ONH may also be a contributing factor.

We also sought to determine whether the iron overload in RPE due to loss of liver-produced Hepc is associated with reduced bisretinoid levels. We measured bisretinoids in LS-Hepc^−/−^ mice at 6 months of age using the noninvasive qAF approach. qAF intensity in LS-Hepc^−/−^ mice (0.88 qAF units) *versus* wild-type mice (0.56 qAF units) did not reach a statistically significant difference at the age of 6 months (Fig. 1*E*). NIR-AF that originates primarily from RPE melanin was measured in a circular area centered on the ONH and having a diameter of 3 mm. There was no difference between wild-type and LS-Hepc^−/−^ mice. At 12 months of age, however, while peaks attributable to the bisretinoids A2E, iso-A2E, A2-glycerophosphoethanolamine (A2-GPE), A2-dihydropyridine-phosphatidylethanolamine (A2-DHP-PE), and all-*trans*-retinal dimer phosphatidylethanolamine (atRAL-di-PE) were observed in HPLC chromatograms generated with extracts of wild-type mice, these peaks were greatly diminished or undetectable in the chromatograms acquired from LS-Hepc^−/−^ mice (Fig. 1*F*). HPLC quantitation of A2E in the LS-Hepc^−/−^ mice revealed a 63% reduction in A2E relative to the wild-type mice (*p* < 0.01, unpaired two-tailed t-test).

### Emission spectra

**Figure 2:**
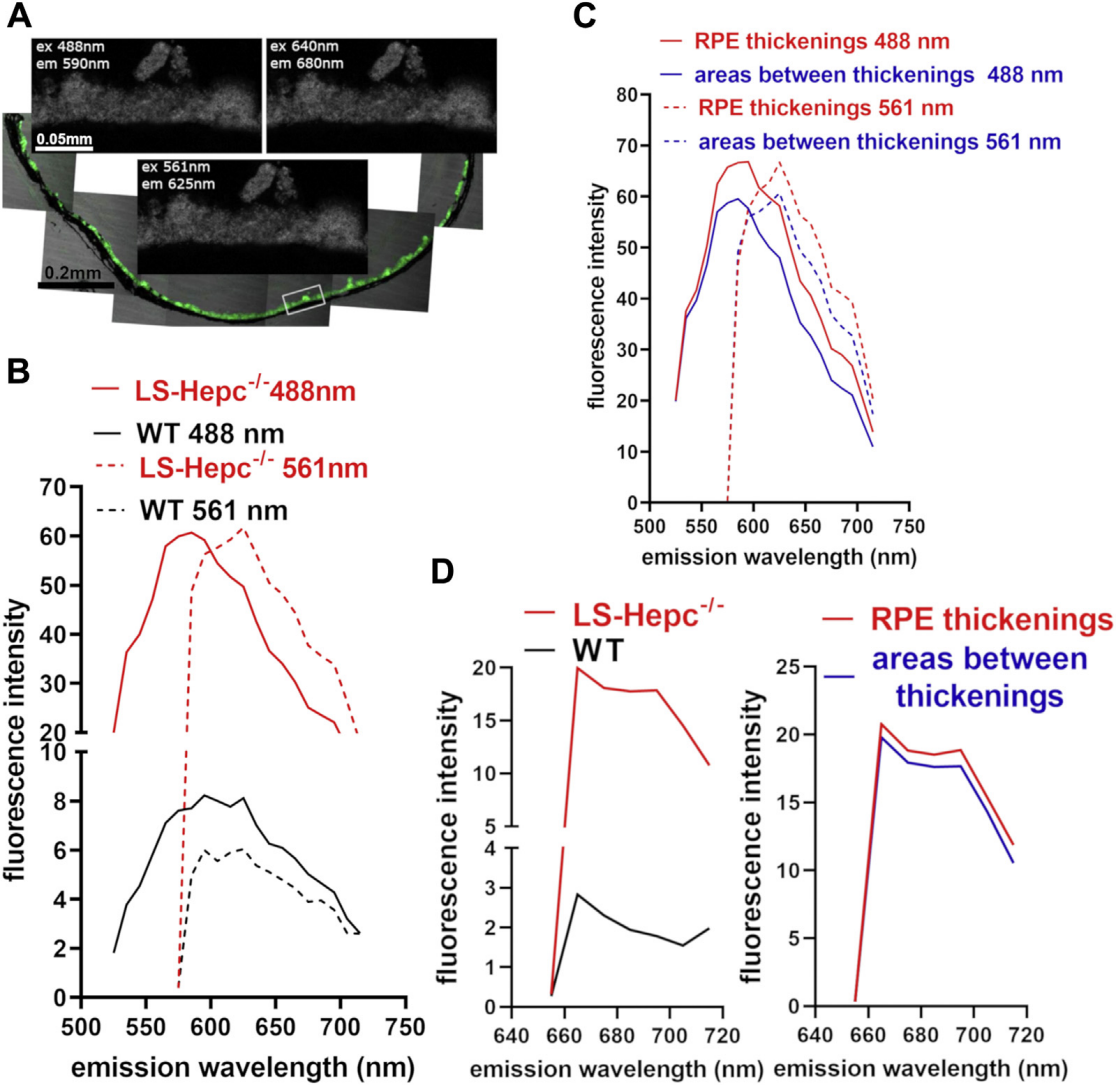
**Emission spectra of liver-specific hepcidin knockout (LS-Hepc**^**−/−**^**) mice.***A*, representative fluorescence micrographs of RPE layer in a male LS-Hepc^−/−^ mouse. The area used to sample spectra is indicated (*white box*). Note thickened foci in RPE monolayer. *B*, emission profiles recorded from the RPE monolayer of mutant and control mice at 488 and 561 nm excitation. Traces represent the average of six recordings at 10 nm intervals (three eyes each of mutant and control, two locations in each eye). *C*, Comparison of spectra recorded from abnormal RPE thickenings (as in *A*) and areas between RPE thickenings in LS-Hepc^−/−^ mice. *D*, spectra recorded at 640 nm excitation in LS-Hepc^−/−^*versus* control mice (*left*) and at locations of RPE thickenings *versus* areas in between in LS-Hepc^−/−^ mice (*right*).

The fluorescence emission generated from bisretinoid lipofuscin in the RPE of LS-Hepc knockout mice (age 11, 13, 14.5 months) and in wild-type controls (age 13 months) was recorded at 488, 561, and 640 nm (Fig. 2). The emission maximum recorded from both the mutant and wild-type eyes with 488 nm excitation was 590 nm. The spectra were also similar in shape as was the spectral width at half-maximal intensity (50 nm and 60 nm, LS-Hepc^−/−^ and WT, respectively). At 561 nm excitation the peak emissions recorded from the LS-Hepc^−/−^ and WT mice were red-shifted to 625 nm and the spectral width was narrower (40 nm in both mutant and WT mice) (Fig. 2*B*). The spectra recorded with 640 nm excitation had little structure within the emission range studied (Fig. 2*D*).

The intensity difference between the mutant and wild-type mice was pronounced. The peak intensity was sevenfold and tenfold greater in the LS-Hepc^−/−^ mice at 488 nm and 561 nm excitation, respectively (Fig. 2*B*). In mutant mice, auto-fluorescence emission was more intense when recorded at the RPE thickenings *versus* areas between the thickenings (Fig. 2, *C* and *D*).

### Photo-Fenton process

**Figure 3:**
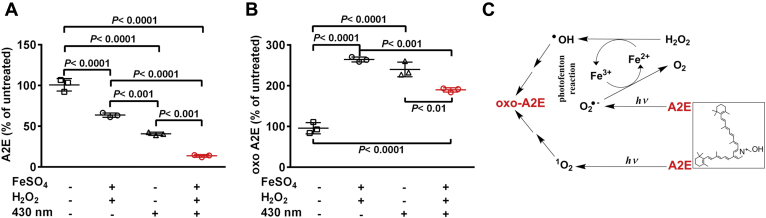
**Synergistic effects of iron (Fe), hydrogen peroxide (H**_**2**_**O**_**2**_**), and light (*hv*) in the oxidation of the bisretinoid A2E.** Photo-Fenton reaction *A* and *B*, UPLC quantitation of A2E and oxidized-A2E (oxo-A2E) after incubation with FeSO_4_ and H_2_O_2_, with and without 430 nm irradiation. Means ± SD, *n* = 3. *p* values determined by two-way ANOVA, Tukey’s multiple comparison test. *C*, proposed mechanism. Hydroxyl radical (^•^OH) generated by Fe^2+^ and H_2_O_2_ (Fenton reaction) oxidizes A2E and produces Fe^3+^. Fe^2+^ is replenished by donation of an electron from superoxide anion (O_2_^•−^); the latter forms by irradiation of A2E. Photosensitization of A2E generates O_2_^•−^ and singlet oxygen ^1^O_2_ that also oxidize A2E.

The Fenton reaction can efficiently generate highly reactive hydroxyl radicals (^•^OH) through the decomposition of H_2_O_2_ by Fe^2+^ (H_2_O_2_/Fe^2+^). We have shown that bisretinoids are oxidized both by photooxidative processes (12, 13, 21) and by iron-mediated formation of hydroxyl free radical *via* the Fenton reaction (11, 22). We note, however, that the production of the hydroxyl radical *via* the Fenton reaction depends on continued availability of Fe^2+^ (ferrous iron). Thus we evaluated the ability of visible light to potentiate the oxidation of bisretinoid, presumably by the photoreduction of Fe^3+^ (ferric iron) to Fe^2+^ (ferrous iron) (photo-Fenton process) (14, 23). When A2E was incubated with FeSO_4_ and H_2_O_2_ at 37 °C for 30 min and then irradiated with 430 nm light (15 s) at an intensity of 290.6 lux (H_2_O_2_/Fe^2+^/light), UPLC quantitation of A2E revealed a 86% reduction in A2E as the substrate (*p* < 0.0001, one-way ANOVA, and Tukey's multiple comparisons test) (Fig. 3*A*). Incubation of A2E with FeSO_4_ and H_2_O_2_ in the absence of light was associated with a less pronounced decline (36%) in A2E (*p* < 0.0001, one-way ANOVA, and Tukey's multiple comparisons test), and A2E was decreased by 59% with irradiation at 430 nm alone (*p* < 0.0001, one-way ANOVA and Tukey's multiple comparisons test). Oxidation of A2E was also demonstrated by the detection of oxidized A2E (*m/z* 592 + 16). After irradiation at 430 nm the proportion of oxidized A2E in the sample increased 2.4 times compared with unirradiated A2E (*p* < 0.0001, one-way ANOVA, and Tukey's multiple comparisons test) while irradiation in the presence of FeSO_4_ and H_2_O_2_ was associated with a 1.9 times increase in oxidized A2E (*p* < 0.0001, one-way ANOVA, and Tukey's multiple comparisons test) (Fig. 3*B*).

### Iron is elevated in RPE of *Abca4*^−/−^ mice

**Figure 4:**
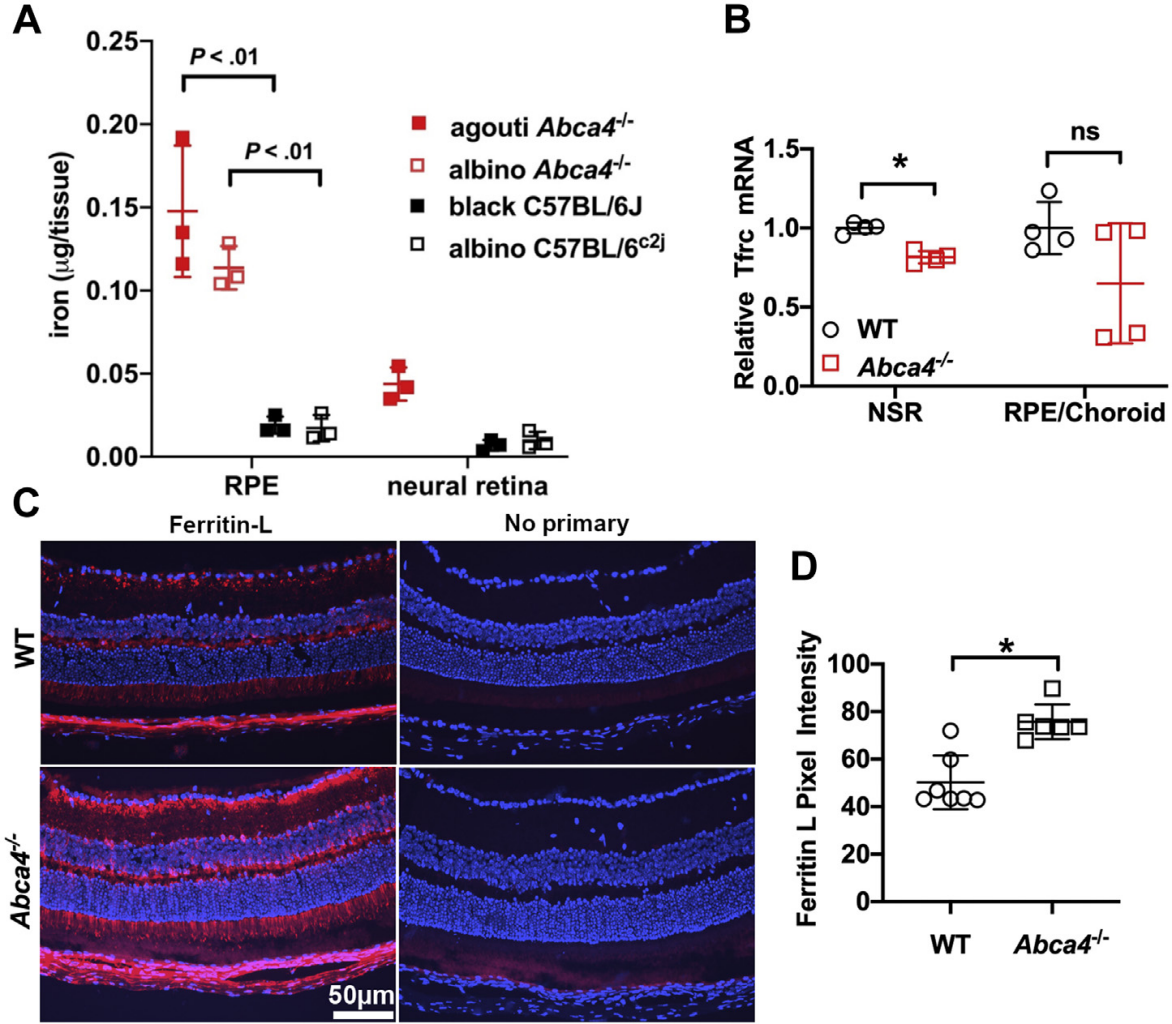
**Iron, ferritin light chain (ferritin-L), and transferrin receptor (Tfrc) quantitation in neural retina (NR) and retinal pigment epithelium (RPE) of *Abca4***^−/−^**and wild-type mice.***A*, iron quantitation by ferrozine-based colorimetric assay. Values are means ± SD; two eyes per sample; *n* = 3. *p* values were determined by one-way ANOVA and Tukey’s multiple comparison test. *B*, transferrin receptor (Tfrc) mRNA expression in mouse neural retina and RPE/choroid. Albino *Abca4*^−/−^ mice, age 8 months. Tfrc was significantly downregulated in the neurosensory retina. Values are means ± SD of one eye per mouse; *n* = 4. *p*-values were determined by two-tailed t-test. WT *versus Abca4*^−/−^: NSR (neurosensory retina), ∗*p* < 0.01; RPE *p* > 0.05. *C*, Ferritin-L expression detected by immunohistochemistry in retinal cryosections. Ferritin-L (FTL) labeling is present in the neurosensory retina and RPE of both wild-type (WT) and *Abca4*^−/−^ mice age 8 months. *D*, quantification of the mean pixel intensity in the RPE and photoreceptor layers indicates significantly increased ferritin-L positivity in the *Abca4*^−/−^ mice. Values are means ± SD of one cryosection per mouse; *n* = 7. WT *versus Abca4*^−/−^: ∗*p* < 0.001, two-tailed *t*-test. Groups were composed of female and male mice and were gender-matched.

Within cells, the majority of iron is stored in ferritin, a cytosolic protein (24). We measured iron levels in the RPE and neural retina of *Abca4*^−/−^ and wild-type mice both pigmented and albino using a colorimetric assay based on ferrozine, a water-soluble chelator of Fe^2+^ (25, 26) (Fig. 4*A*). Iron levels were higher in RPE than in neural retina, and this difference was significant in agouti *Abca4*^−/−^ and albino *Abca4*^−/−^ mice (*p* < 0.0001; for agouti *Abca4*^−/−^, *p* < 0.05 for albino *Abca4*^−/−^ two-way ANOVA, Tukey’s multiple comparison test) (Fig. 4*A*). It is especially noteworthy that iron levels in RPE were higher in agouti and albino *Abca4*^−/−^ mice than in RPE cells harvested from wild-type C57BL/6J and C57BL/6^c2j^ mice (*p* < 0.0001, for agouti *Abca4*^−/−^, *p* < 0.001 for albino *Abca4*^−/−^two-way ANOVA, Tukey’s multiple comparison test) (Fig. 4*A*).

While iron is considered to also be stored in association with melanin (24), our measurements of iron levels were not significantly different in the RPE of albino C57BL/6^c2j^ mice as compared with black-coated C57BL/6J mice (*p* > 0.05 two-way ANOVA, Tukey’s multiple comparison test) (Fig. 4*A*).

### Ferritin and transferrin receptor in *Abca4*^−/−^ mice

Further indication of retinal iron overload was provided by measuring Tfrc mRNA levels in isolated neural retina and RPE by real-time qPCR. Relative Tfrc mRNA levels were significantly decreased in the neural retina of *Abca4*^−/−^ mice (*p* < 0.001) and decreased but not significantly in the RPE of the *Abca4*^−/−^ mice (Fig. 4*B*). This is additional evidence of increased intracellular iron levels in the RPE and NSR of the *Abca4*^−/−^ mice.

To further examine iron accumulation in the retina of *Abca4*^−/−^ mice, we assessed retinal levels of the iron storage protein ferritin light chain (ferritin-L) using immunohistochemistry. The RPE and photoreceptor layers stained positively for ferritin-L in both the wildtype and *Abca4*^−/−^ 8-month-old mice (Fig. 4*C*) but quantitation shows significantly increased ferritin-L positivity in the RPE and photoreceptor layers of the *Abca4*^−/−^ mice (*p* < 0.001) (Fig. 4*D*).

**Figure 5:**
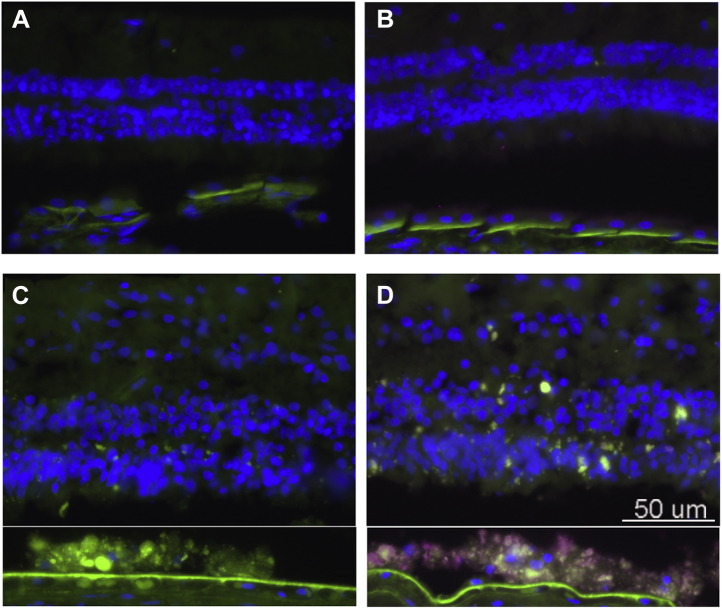
**Ferritin is increased in RPE of patient diagnosed with recessive Stargardt disease (STGD1).** Fluorescence micrographs of human retinal sections immunostained with mouse anti-ferritin-L. Ferritin, *purple*; *green*, RPE lipofuscin autofluorescence. *A*, healthy control eye, no primary antibody. *B*, healthy control, anti-Ferritin L. *C*, STGD1 control: no primary antibody. *D*, STGD1, anti-Ferritin-L immunostaining. Note that the neural retina was detached from the RPE during tissue processing. Thus the RPE-region of the image was moved closer to the neural retina using Photoshop. *Green*, RPE lipofuscin autofluorescence.

By immunocytochemical staining using mouse antihuman ferritin-light chain, we also observed increased ferritin, (indicative of increased iron) that was localized to RPE in cryostat sections of the eyes from patients diagnosed with STGD1 (Fig. 5).

### qAF and bisretinoid quantitation in DFP-treated BALB/cJ mice

**Figure 6:**
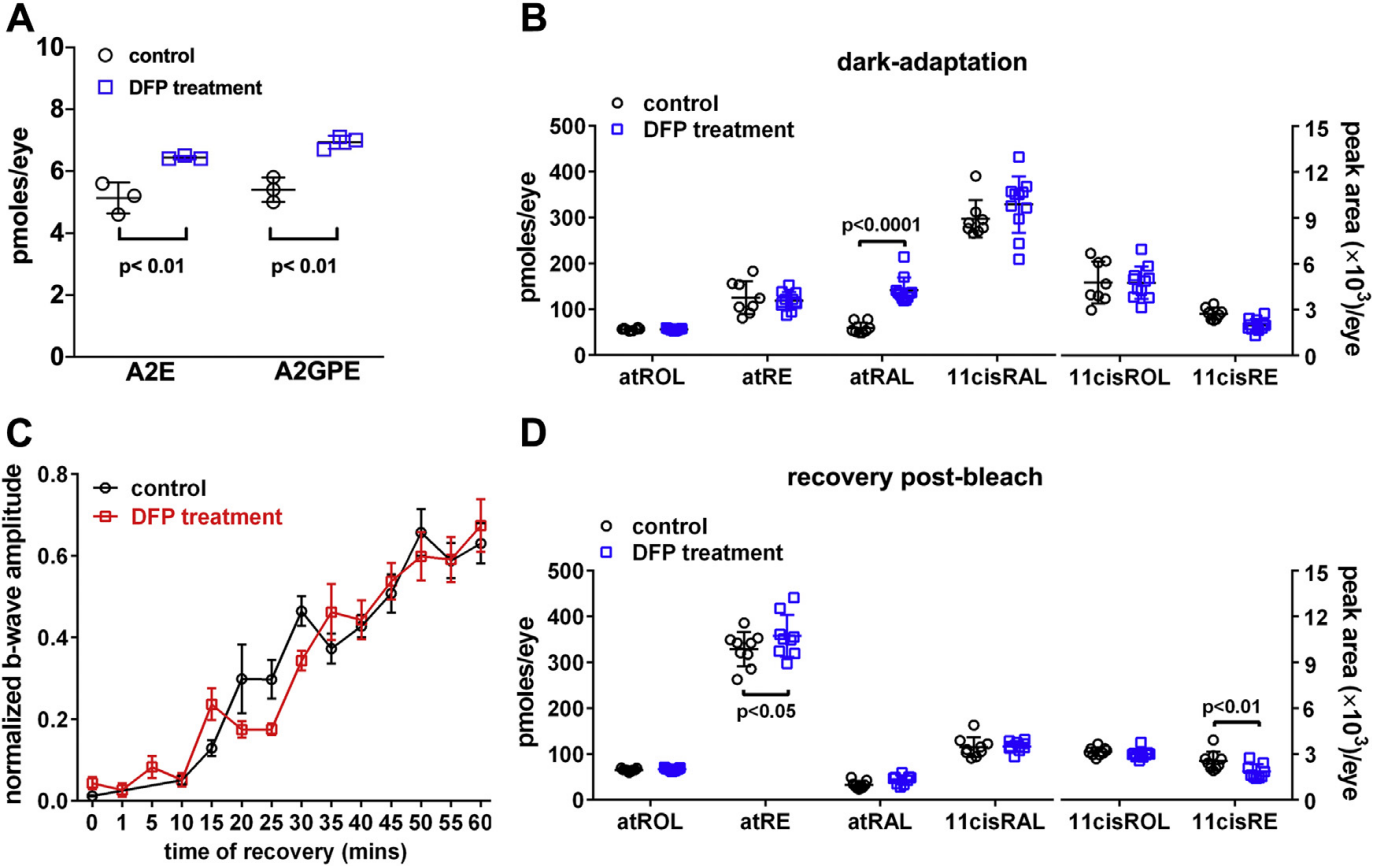
**Analysis in mice treated with the iron chelator deferiprone (DFP) (1 mg/ml in drinking water).***A*, chromatographic quantification of the bisretinoids A2E and A2-GPE in BALB/cJ mice (age 4 months; male) treated with the iron chelator deferiprone for 2 months; 4 eyes/sample; *n* = 3. *p* values determined by one-way ANOVA and Tukey’s multiple comparison test. *B*, quantitation of retinoids in dark-adapted BALB/cJ mice treated with DFP from age 1 to 2 months; all-*trans*-retinol (atROL) all-*trans*-retinyl palmitate (atRE), all-*trans*-retinal (atRAL), 11-*cis*-retinal (11cisRAL), 11-*cis*-retinol (11-cisROL), 11-*cis* retinyl-palmitate (11cisRE) are presented as picomoles per eye; 11-*cis*-retinol and 11-*cis*-retinyl ester are presented as peak area/per eye. Mean ± SD of 8 to 11 samples (female). *p* values determined by ANOVA and Sidak’s multiple comparisons test. *C*, recovery of ERG b-wave amplitudes in BALB/cJ mice (age 3 months; male) treated with DFP for 1 month. B-wave amplitude is plotted as a function of time after the bleaching exposure. Responses were normalized to the prebleach dark-adapted baseline and fit arbitrarily by single exponential curves. SE bars are shown. *n* = 4 to 12. *D*, DFP- and untreated-mice (age 3 months) were dark adapted overnight, exposed to bleaching light, and retinoid recovery was measured after 1 h in the dark (nine samples, one eye/sample) (male). Means ± SD, *p* values determined by two-way ANOVA, Sidak’s multiple comparison test.

The FDA-approved iron chelator DFP is orally absorbed and cell-permeable and known to reduce serum iron and intracellular iron levels in the retina (11, 27, 28). DFP not only binds iron, it also oxidizes Fe^2+^ to Fe^3+^, thus impeding the effects of Fe^2+^ the major catalyst of free radical damage to cells (29). We treated BALB/cJ mice with oral DFP from age 2 months to 4 months and observed a 25% increase in the bisretinoid A2E and a 28% increase in A2-GPE (Fig. 6*A*). We also measured bisretinoid noninvasively (30) by employing *in vivo* fundus autofluorescence after only 1 month of DFP treatment. With this shorter duration of treatment, the difference in fluorophore accumulation between DFP-treated and untreated mice was less pronounced (*p* > 0.05, two-tailed *t*-test). We surmised that the changes in levels of bisretinoid were indicative of DFP-mediated reduction in endogenous iron-associated degradation of A2E and A2-GPE.

### DFP-treated mice: measurement of retinoids and recovery of ERG amplitudes after visual pigment bleaching

The rate limiting enzyme of the visual cycle is Rpe65, the isomerohydrolase essential for the conversion of all-*trans*-retinyl ester to 11-*cis* retinaldehyde (15). Rpe65 has an iron binding domain and the catalytic activity of Rpe65 is dependent on the availability of iron (6). Thus we examined for an effect of iron chelation on steady-state levels of retinoids in dark-adapted BALB/cJ mice (age 2 months) that were treated with DFP in drinking water for 1 month. BALB/cJ mice were used for these experiments because the latter mice carry wild-type Rpe65 (Rpe65-Leucine 450) while C57BL/6J mice carry the methionine variant. No differences in DFP-treated *versus* untreated mice were observed for 11-*cis*-retinal, all-*trans*-retinol and all-*trans*-retinyl ester, 11-*cis*-retinol, and 11-*cis*-retinyl ester. However, inexplicably all-*trans*-retinal was more abundant in the DFP-treated mice (Fig. 6*B*). We also measured the recovery of 11-*cis*-retinal levels 1 h after exposure to bleaching light in dark-adapted mice (BALB/cJ, age 3 months). UPLC measurement of retinoids in mice maintained in darkness for 1 h after the bleach revealed that in mice treated with DFP for 1 month, 11-*cis*-retinal, 11-*cis*-retinol, and all-*trans*-retinol were not different than in the untreated BALB/cJ mice (*p* > 0.05, two-way ANOVA, and Sidak’s multiple comparison test). In the DFP-treated mice, there was, however, a small increase in all-*trans*-retinyl ester (8% increase) and a 27% decrease in 11-*cis*-retinyl ester, both of which reached statistical significance (*p* < 0.05) (Fig. 6*D*).

For an additional test of iron chelation on the visual cycle, we also compared DFP-treated and untreated mice in terms of the time to recovery of dark-adapted ERG b-wave amplitudes after photobleaching (90% bleach). The DFP-treated and nontreated dark adapted mice exhibited similar postbleach recovery of the b-wave amplitude (Fig. 6*C*).

## Discussion

STGD1 is an early onset monogenic disease caused by mutations in the *ABCA4* gene. The ABCA4 protein is located in the outer segments of rod and cone photoreceptor cells and assists in the removal of retinaldehyde by flipping *N*-retinylidene-phosphatidylethanolamine (NRPE), the Schiff base that forms by reaction of retinaldehyde with phosphatidylethanolamine in the disc membrane (31, 32). ABCA4 insufficiency in STGD1 allows for reaction of NRPE with a second molecule of retinaldehyde thereby leading to the formation of bisretinoid. The latter fluorophores are subsequently transferred to RPE within phagocytosed outer segment discs. Evidence available from mouse models and clinical studies of ABCA4-related disease indicates that accelerated bisretinoid formation is the cause of retinal degeneration in STGD1 (22, 33, 34, 35, 36). Moreover, outer nuclear layer width, an indicator of photoreceptor cell viability, is reduced in *Abca4*^−/−^ mice by 8 to 9 months of age and is progressive (37). It is thus notable that DFP treatment of *Abca4*^−/−^ mice attenuates this thinning (11). Here we have also observed that quantities of iron measured spectrophotometrically are elevated in *Abca4*^−/−^ mice relative to wild-type mice. Moreover, in the retina of *Abca4*^−/−^ mice, the iron storage protein ferritin-L was more abundant than in wild-type mice, and mRNA encoding the transferrin receptor, which serves as iron importer, was downregulated. These changes in transferrin receptor mRNA reflect elevated cellular iron levels; when labile (free) iron levels are elevated transferrin receptor is reduced (38). Why and how iron levels increase in tandem with other disease features of STGD1 requires investigation. Besides reflecting levels of iron stores, ferritin is an acute phase protein that undergoes changes in concentration in response to inflammatory processes (39, 40). This issue is perhaps of significance given that products of bisretinoid oxidation are implicated in the activation of the complement system (41, 42). This is a factor that we are addressing in *Abca4*^−/−^ mice.

Of the iron regulatory proteins, hepcidin provides systemic control by inducing degradation of the iron exporter ferroportin, thereby limiting extracellular efflux of iron and causing intracellular iron retention (8). Although hepcidin is primarily produced by hepatocytes, a few other cell types such as in the retina (43) can also produce the protein. Hepcidin serves as central regulator of systemic iron by controlling the release of iron from liver stores, intestinal enterocytes, and macrophages involved in recycling erythrocytes (44). It has been shown previously that systemic Hepc knockout leads to high circulating iron levels as a result of unrestricted iron absorption from the diet. As a consequence, iron accumulates in the retina leading to retinal degeneration in mice by 18 months of age (9). Subsequently it was found that while LS-Hepc^−/−^ mice retained the function of hepcidin in the retina, iron levels were elevated in the blood at age 3 and 12 months. RPE cells also exhibited elevated iron at age 6 months and profoundly reduced Tfr mRNA at 3, 6, and 12 months (16).

Here we observed that local retinal iron regulation in the setting of chronic systemic iron overload was not sufficient to ward off iron-induced oxidative processes in RPE. Unlike experimental models that employ nonspecific exogenous oxidants, in these mechanistic studies we monitored the oxidation of bisretinoid, an *in vivo* system recognized for its photooxidative activity (12, 13, 22, 45). We showed previously that iron and H_2_O_2_ can also oxidize bisretinoid, presumably through generation and oxidation by hydroxyl radical (^•^OH) (11). In the current work, iron-mediated degradation of bisretinoid was realized by a decrease in HPLC-quantified bisretinoid. These processes of oxidation and degradation of bisretinoid can occur in the presence of both light and Fenton chemistry (11, 22) and likely mediate chronic and accumulative cellular and molecular damage. The polyene arms of bisretinoid fluorophores can be oxidized by singlet oxygen, hydroxyl free radicals, and probably superoxide anion. The molecule subsequently fragments into aldehyde- and dicarbonyl-bearing products (12, 13) that include the dicarbonyls methylglyoxal (MG) and glyoxal (GO) (12, 13). These dicarbonyls modify proteins and have been described in sub-RPE drusen (46, 47). This photodegradative process is responsible for the lower bisretinoid levels measured in albino *versus* black mice and in light- *versus* dark-reared mice (22) and may be the reason why early and intermediate AMD are associated with lower fundus autofluorescence intensity (measured as quantitative fundus autofluorescence, qAF) in central retina (48).

We have shown previously that while individual bisretinoids that constitute the lipofuscin fluorophores of RPE can be excited at a range of excitations varying from 440 nm to 510 nm, all of these fluorophores emit fluorescence centered on 600 nm (18, 20, 49, 50, 51, 52). Consistent with our previous work (52) and with studies of fundus autofluorescence (53), we observed here that the fluorescence emission from lipofuscin bisretinoid in the RPE of LS-Hepc^−/−^ mice was shifted toward longer wavelengths as excitation wavelength was increased; additionally the spectral width was reduced. These features reflect fluorescence generation from the complex mixture of bisretinoids that we have described (51, 52, 54, 55). Indeed, the contributions that individual bisretinoids make to fundus autofluorescence can be influenced not only by their excitation and fluorescence spectra but also by their abundance and fluorescence efficiency (52).

The finding of a reduction in HPLC-quantified bisretinoid in LS-Hepc^−/−^ mice concurrent with an increase in fluorescence emission intensity at first glance seems to be a contradiction. By way of understanding, we note that the fluorescence intensities of some oxidized forms of bisretinoid can be many-fold greater than the parent molecule. For instance, addition of one and two oxygen atoms on the short-arm of A2E increases fluorescence efficiencies by as much as 12-fold (55). The addition of the oxygens also increases the polarity of the molecule, conferring a shorter retention time in a reversed-phase HPLC column and thus a reduction of the A2E chromatographic peak. Other bisretinoids behave similarly. These events are consistent with an increase in overall fluorescence emitted from the bisretinoid mixture despite a reduction in the amount of each parent molecule. The extent to which these oxidized bisretinoids accumulate before fragmentation is the subject of further study.

By employing A2E as the substrate in *in vitro* experiments, we also tested the impact of photo-assisted Fenton reaction using visible light together with H_2_O_2_ and Fe^2+^. We observed that the oxidation of A2E was greater when iron, H_2_O_2_, and light were provided in the reaction mixture as compared with the presence of H_2_O_2_ and Fe^2+^ (in darkness) or light alone. These experiments demonstrated that light in the presence of the photosensitizer A2E can potentiate the Fenton reaction probably due to reduction of Fe^3+^ to Fe^2+^ by superoxide (O_2_•^−^) (11, 56, 57) (Fig. 3*C*). Superoxide generated by visible light-irradiation of A2E (57) would donate one electron to reduce Fe^3+^ to Fe^2+^ (58). This illustration of synergy among the Fenton reaction, photosensitization of A2E, and redox conversion of the Fe^+2^ and Fe^+3^ oxidation states is significant to the retina since unlike most tissues, the retina is exposed to visible light. Moreover, since Fe^2+^ can be regenerated in the presence of visible light, even a small amount of Fe^2+^ may have a sizable effect.

As in a previous report (11), we demonstrated in the current study that treatment with the iron chelator DFP is associated with increased bisretinoid levels; we interpret this as being due to reduced iron-mediated bisretinoid degradation. Bisretinoids form as a by-product of the visual cycle and reduced Rpe65 activity suppresses the formation and accumulation of bisretinoid fluorophores (59). Since RPE65, the rate-limiting enzyme of the visual cycle, has an iron binding domain (6), we tested for an effect of DFP-chelation on RPE65 activity. Interestingly, recovery of b-wave amplitude and regeneration of 11-*cis*-retinal 1 h after chromophore bleaching were not impaired in DFP-treated *versus* untreated mice. Nonetheless, an increase in all-*trans*-retinyl ester and a decrease in 11-*cis*-retinyl ester were observed. The latter changes are consistent with an effect on RPE65 even in the absence of a difference in the visual chromophore 11-*cis*-retinal. Even so, iron chelation of Rpe65 cannot explain the change in bisretinoid levels since if iron deficiency conferred by DFP was to affect RPE65 activity, the impact would be realized as reduced bisretinoid, not an increase as we observed. The upshot of this observation is that DFP therapy in the context of iron overload can be employed without adverse effects on visual sensitivity.

Taken together our findings indicate that iron chelation can attenuate toxic bisretinoid oxidative degradation (Fig. 6) while iron overload can promote this activity (Fig. 1). While we showed that iron levels are elevated in the retina afflicted with STGD1, dysregulation of iron may also be a secondary factor in other retinal disorders. For instance, transferrin is upregulated in patients with AMD (60) and zinc deferoxamine has been shown to enhance photoreceptor survival in the rd10 model of retinitis pigmentosa (61). Similarly, DFP can partially protect WT mouse retinas from light damage (27) that has also been linked to the phototoxicity of bisretinoid (37). Thus iron chelation may be retina-protective in diseases involving bisretinoid toxicity including STGD1.

## Experimental procedures

### Mice

Albino *Abca4*^−/−^ (Rpe65 Leu450) and agouti *Abca4*^−/−^ (Rpe65 Leu450) were bred in-house. C57BL/6J (Rpe65 450Met variant), C57BL/6^c2j^ (Rpe65 450Met variant), and BALB/cJ (Rpe65 Leu450) were purchased from Jackson Laboratory (Bar Harbor, ME, USA) and were all gender-matched. Liver-specific (LS) hepcidin knockout mice (LS-Hepc^−/−^) (Rpe65–450Met gender-matched with controls) had been generated as described; the null mutation of hepcidin in the liver was confirmed by real-time quantitative PCR (62). Animal protocols were approved by the Institutional Animal Care and Use Committees of Columbia University and University of Pennsylvania and complied with guidelines set forth by the Association for Research in Vision and Ophthalmology (ARVO) Animal Statement for the use of animals in ophthalmic and vision research. All mice were fed a standard laboratory diet containing 300 ppm iron and given free access to water. They were maintained in a temperature-controlled room at 21 to 23 °C under cyclic light (12 h: 12 h light–dark cycle) during the experiments. Mice were negative for the rd8 and rd1 mutations.

### Treatment with iron chelator

BALB/cJ mice (Rpe65 Leu450) received the iron chelator DFP (Ferriprox) in drinking water (1 mg/ml) for the indicated durations. The intake of DFP water was approximately 3 ml per day.

### Quantitative high-performance liquid chromatography (HPLC) and ultra-performance liquid chromatography (UPLC)

Mouse eyecups (2–4 eyes/sample as indicated) were homogenized, extracted in chloroform/methanol (2:1), and analyzed for bisretinoids (A2E, iso-A2E, A2-DHP-PE, atRAL di-PE) by reversed-phase HPLC using an Alliance System (Waters, Milford, MA) and Atlantis dC18 column or Waters Acquity UPLC-MS system and Acuity BEH phenyl column (A2GPE) (Waters, Milford, MA) as described (22). Molar quantities per eye were calculated by comparison with synthesized standards. The pyridinium bisretinoid A2E and its *cis* isomer, iso-A2E, were measured separately and summed (A2E/iso-A2E).

For retinoid quantification, mouse eyecups (1 eye/sample) were homogenized and derivatized using *O*-ethylhydroxylamine (63). Retinal *O*-ethyloxime was extracted with hexane and resuspended in acetonitrile. The Waters Acquity UPLC-MS system was used with a CSH C18 column (1.7 μm, 2.1 × 100 mm; Waters, Milford, MA) and gradients of water (A) and acetonitrile (B) with 0.1% of formic acid as follows: beginning at 60% B, holding for 5 min, followed by a linear increase to 70% B over 55 min, followed by a linear increase to 100% B over 10 min and holding for 20 min (flow rate of 0.3 ml/min) (64).

### Iron assay

Mouse eyes were dissected to isolate and separate RPE and neural retina. The tissues were homogenized in 200 μl distilled water and incubated with 3% trichloroacetic acid for 30 min at 4 °C. The mixture was centrifuged at 6000 rpm for 15 min, and the supernatant was used as a sample. Iron concentration was determined using JaICA iron assay (ferrozine chromogenic method) (Nikken SEIL Co, Ltd, Japan) with measurement of absorbance at 560 nm in a plate reader. Iron concentration was determined by comparison with the absorbance of a known standard.

### Fundus imaging

Mice were anesthetized, pupils were dilated, the cornea was lubricated as previously described (30). Fundus AF images (55° wide field lens; 0.98-mm detection pupil) at 488 nm and 790 nm excitation were obtained with a confocal scanning laser ophthalmoscope (Spectralis HRA; Heidelberg Engineering, Heidelberg, Germany) with laser power set at approximately 280 μW and sensitivity at 100 and 105, respectively, after visual pigment was bleached for 20 s. Nine successive frames were acquired at 488 nm excitation with the high-speed mode, and frames were saved in nonnormalized mode. A mean of 100 frames was obtained at 790-nm excitation with high-resolution automatic real-time mode and resized with Photoshop CS4 (Adobe Systems, Inc, San Jose, CA, USA) to 768 × 768 pixels, the same as high-speed mode images. Near-infrared reflectance images (NIR-R) (820 nm) were also acquired.

SW-AF intensities at 488-nm excitation were measured (quantitative fundus autofluorescence, qAF) by acquiring mean gray levels (GLs) in eight predefined segments around the optic disc as previously described (30). qAF was calculated by normalization to the GL of the reference after subtraction of zero light (GL_0_) and inclusion of a reference calibration factor (4, 5). Fluorescence intensities at 790 nm were calculated by subtracting the minimal GL of the optic nerve head measured by ImageJ software (http://imagej.nih.gov/ij/; provided in the public domain by the National Institutes of Health, Bethesda, MD, USA).

### Immunofluorescence

Deidentified human tissue from the Foundation Fighting Blindness was exempt from IRB approval. Eyecups were dissected from globes fixed in 4% paraformaldehyde after removing the cornea and lens. Eyecups were dehydrated in 30% sucrose overnight and embedded in optimal cutting temperature compound (OCT; Tissue-Tek, Sakura Finetek, Torrance, CA). Immunofluorescence was performed on 10-μm-thick cryosections, as previously described (65). The primary antibody used was rabbit anti-light ferritin (E17) at 1:2500 dilution (gift of Maura Poli and Paolo Arosio, University of Brescia, Italy). Fluorophore-labeled anti-rabbit antibodies (Jackson ImmunoResearch Laboratories, West Grove, PA) were used to detect primary antibody reactivity. Control sections were treated identically, but with omission of the primary antibody. Sections were analyzed by fluorescence microscopy with identical exposure parameters using Nikon Elements software (Melville, NY). Immunoreactivity was quantified by measurement of the mean pixel intensity within the RPE and photoreceptor layers of each photomicrograph using ImageJ software (http://imagej.nih.gov/ij/).

### Quantitative real-time PCR

RNA isolation was performed on neurosensory retina and RPE/choroid samples according to the manufacturer’s protocol (RNeasy Kit; Qiagen, Valencia, CA). Reverse transcription reagents (Taqman; Applied Biosystems, Darmstadt, Germany) were used per protocol to synthesize cDNA. Quantitative detection of the mRNA encoding transferrin receptor (Tfrc, Mm00441941_m1) (9) was performed using gene expression assays (TaqMan; Applied Biosystems), qPCR instrumentation (ABI Prism 7500 Sequence Detection System; Applied Biosystems), *Gapdh* as an internal control (Mm99999915_g1), and the ΔΔC_T_ method. All reactions were performed using four biological replicates and three technical replicates.

### Emission spectra

Emission spectra were recorded from the RPE monolayer in cryostat sections of mouse eyes using confocal laser scanning fluorescence microscope (Nikon A1plus DUV-B spectral confocal microscope) with a 60×/1.4 ApoTIRF oil lens objective. The pinhole was set at maximum diameter. Laser excitation was 488, 561, and 640 nm; the beam was directed through an 80/20 beam splitter and data were recorded in 10 nm increments. Emission data were adjusted to pixel size (field size, 512 pixels wide; 0.377 μm per pixel) and laser power (488 nm, 2.3 mW; 561 nm, 3 mW; 640 nm, 5 mW).

### Electroretinography

BALB/cJ mice (six mice) (Rpe65-Leu450) were given water medicated with DFP from age 2 to 3 months. DFP-treated (six mice) and untreated (six mice) were dark-adapted and anesthetized with an intraperitoneal injection of a mixture of ketamine, xylazine, and acepromazine. Pupils were dilated with topical phenylephrine hydrochloride and tropicamide. ERG recordings were obtained with a Celeris rodent ERG system (Diagnosys, Lowell, MA) from a corneal electrode positioned with 2.5% hypromellose (Akom, Lake Forest, IL). The mouse rested on a precisely controlled integrated heater that maintains body temperature at 35 to 37 °C. After recording dark-adapted baseline responses to a white light stimulus of 1 cd s/m^2^ at 0.2 Hz, the eyes were exposed to 1000 cd s/m^2^ white light for 5 min to bleach rhodopsin (90%). ERG responses to 1 cd s/m^2^ white light were recorded immediately after bleaching, at 1 min and 5 min post bleaching, and then every 5 min for amplitude recovery until 60 min post bleaching. Three successive responses were averaged using interstimulus interval of 10 s; the latter was chosen to ensure that dark adaptation was maintained.

### Photo-Fenton reaction

Synthesized A2E (50 μM) (18) was incubated with FeSO_4_ (200 μM) and H_2_O_2_ (500 μM) for 30 min at 37 °C, after which the reaction mixture was irradiated with blue light (430 ± 20 nm; 1 mW/cm^2^) for 15 s. These samples were measured by Waters Acquity UPLC system (Waters, Milford, MA). UPLC analysis was performed using an XBridge BEH C18 column (2.5 μm, 3.0 × 50 mm) with monitoring at 430 nm. A2E and its oxidized peaks were separated with water (solvent A) and acetonitrile/methanol = 1:1 (solvent B) with 0.1% formic acid (0–1 min, 70% B, 1–27 min, 70%–98% B, 27–50 min 98% B) at 0.5 ml/min and injection volume of 5 μl.

## Data availability

All of the data are in the article.

## Conflicts of interest

The authors declare that there are no conflicts of interest related to the contents of this article.
